# Integrated Smart Packaging of Modified Silica/Anthocyanin/Nanocellulose for Preservation and Monitoring

**DOI:** 10.3390/foods14111888

**Published:** 2025-05-26

**Authors:** Yu Ren, Jing Guo, Zehao Zhong, Jinjin Chen, Peng Jin, Yonghua Zheng, Zhengguo Wu

**Affiliations:** College of Food Science and Technology, Nanjing Agricultural University, Nanjing 210095, China; renyu@stu.njau.edu.cn (Y.R.); guojing@stu.njau.edu.cn (J.G.); zhongzh@stu.njau.edu.cn (Z.Z.); chenjj@stu.njau.edu.cn (J.C.); pjin@njau.edu.cn (P.J.); zhengyh@njau.edu.cn (Y.Z.)

**Keywords:** nanocellulose, hollow mesoporous silica, anthocyanin, cinnamaldehyde, smart packaging

## Abstract

Smart packaging not only has a preservation effect on food, but can also monitor the change of food quality in real time to ensure food safety. In this study, hollow mesoporous silica loaded with cinnamaldehyde was used as the antimicrobial agent, anthocyanin as the color developer, and nanocellulose as the film matrix, to obtain smart packaging with excellent antimicrobial activity and pH-responsive color development (CBF). Modified silica has a good regulatory characteristic on the release of cinnamaldehyde, and the cumulative release rate of cinnamaldehyde in the NH_2_-HMSN@CA preservative reaches 72% after 7 days. Additionally, the film has good antibacterial properties, with inhibition rates of 82% and 92% against *E. coli* and *S. aureus*, respectively. In addition, the film has good mechanical properties and water vapor permeability. In terms of pH response, the film shows excellent color rendering and good stability. Therefore, the CBF films can be applied to preservation and real-time monitoring of fruits and vegetables, meat, and other food products, which has great potential for intelligent food packaging.

## 1. Introduction

With the further deepening of research in the food industry, multifunctional intelligent food packaging has been developed [1,2,3]. In intelligent packaging, active molecules are added to achieve the preservation effect, maintain the quality of food, and, at the same time, supplemented by indicators and sensors, achieve real-time monitoring of food quality changes to further improve food safety [4,5]. In addition, degradable packaging materials have become a prevailing trend, and polysaccharides and proteins are prominent among the natural materials commonly used [6,7,8,9]. Especially, cellulose has been extensively utilized in the development of food packaging materials due to being widely available and cost-effective [10]. Nanocellulose, produced by reducing cellulose to its nanoscale, exhibits excellent biocompatibility and biodegradability, and its high specific surface area facilitates interactions with other substances. Moreover, its superior mechanical and barrier properties render it an ideal material for green food packaging [11]. Xia et al. [12] prepared a cellulose/alizarin film as a smart indicator film with good mechanical properties and high UV-blocking capacity, and alizarin color change was successfully used to monitor fish freshness. However, nanocellulose has weak antimicrobial properties, which can be enhanced by the addition of antimicrobial substances [13]. Atta et al. [14] added olive oil to nanocellulose films and showed that it exhibited strong antibacterial activity against three bacterial strains and two fungal strains. Therefore, nanocellulose film has prospects for large applications.

Cinnamaldehyde, an essential oil extracted from the roots, leaves, and flowers of cinnamon, has been applied in food packaging for its antimicrobial and antioxidant properties [15]. However, its poor solubility in water, tendency to oxidize in air, and high volatility at elevated temperatures limit its direct application [16,17]. To overcome these limitations, researchers have adopted encapsulation methods to load cinnamaldehyde onto carriers, addressing issues such as volatility and broadening its range of applications. For instance, Mondéjar-López et al. [18] encapsulated cinnamaldehyde in chitosan to produce chitosan films with an encapsulation efficiency of 7%, which demonstrated significant antimicrobial effects over a 20-day period. Hollow mesoporous silica, characterized by its large specific surface area, excellent biocompatibility, and chemical stability, serves as an effective carrier [19,20,21]. Jiang et al. [22] prepared poly(lactic acid) films by loading *nisin* onto mesoporous silica, which can effectively mitigate the release of *nisin* and achieve long-lasting freshness preservation. By functionalizing the silica with amino groups, loading cinnamaldehyde via the Schiff base formation and the hollow cavity enables sustained antimicrobial action [23,24,25].

In addition, during food storage, pH values can undergo significant changes [26]. Therefore, the design of intelligent food packaging that is sensitive to pH variations provides an effective method to monitor changes in food quality. Anthocyanins, widely sourced from the flowers, stems, leaves, and fruits of plants, exhibit distinct colors under different pH conditions, making them suitable as pH indicators in packaging applications [27]. Under strongly acidic conditions (pH < 4), anthocyanins form flavonoid cations, appearing red; at pH values between 4 and 6, deprotonation renders them colorless; in neutral conditions (pH 6–8), the anthocyanins adopt a quinonoid structure, with colors transitioning from pink to purple or blue; and, in alkaline environments (pH > 8), they form chalcone structures that appear yellow [28,29]. Hence, incorporating anthocyanins into packaging systems, such as films and gels, can stabilize their activity and impart pH sensitivity, allowing real-time monitoring of food quality through observable color changes and ensuring food safety [30,31,32,33].

In this study, the nanocellulose-based packaging film (NH_2_-HMSN@CA/BA/CNF) was developed by integrating amino-functionalized hollow mesoporous silica loaded with cinnamaldehyde as the antimicrobial agent, anthocyanin as the color indicator, and nanocellulose as the film matrix. The film presented excellent antimicrobial performance against *E. coli* and *S. aureus*, respectively. In terms of pH responsiveness, the film exhibited distinct color changes: red under acidic conditions, light pink or colorless under neutral conditions, and gray to blue under alkaline conditions. Consequently, the NH_2_-HMSN@CA/BA/CNF film holds significant potential for applications in the packaging of fruits, vegetables, seafood, and meats by providing both fresh-keeping effects and real-time monitoring of food quality.

## 2. Materials and Methods

### 2.1. Materials

Tetraethyl orthosilicate (TEOS) and cetyltrimethylammonium chloride (CTAC) were purchased from Shanghai Macklin Biochemical Co., Ltd., Shanghai, China. 3-aminopropyltriethoxysilane (APTES) and triethanolamine (TEA) was purchased from Sinopharm Chemical Reagent Co., Ltd. (Shanghai, China). TEMPO-oxidation cellulose nanofibers (CNFs) were purchased from Tianjin Woodelf Biotechnology Co. Ltd. (Tianjin, China). Cinnamaldehyde (CA) was purchased from Shanghai Xushuo Biotechnology Co. Ltd. (Shanghai, China). Blueberry anthocyanins (BAs) were purchased from Shanghai Macklin Biochemical Co., Ltd. (Shanghai, China). *Escherichia coli* and *Staphylococcus aureus* were provided by Guangzhou Institute of Microbiology (Guangzhou, China). All other chemicals were of analytical grade.

### 2.2. Synthesis of Nanoparticles

TEOS (6 mL) was added to a 100 mL ethanol/ammonia/water mixture, and the reaction was stirred at 30 °C for 1 h. Then, CTAC (10 g) and TEA (0.5 g) were introduced along with additional tetraethyl orthosilicate, and the mixture was stirred to form mesoporous silica nanoparticles (MSNs). Next, the MSNs were dispersed into a sodium carbonate aqueous solution and stirred for 4 h to form hollow mesoporous silica nanoparticles (HMSNs). Under the 80 °C water bath, APTES (5 mL) was added and stirred for 10 h to obtain NH_2_- HMSN. Finally, a certain amount of cinnamaldehyde was mixed with the NH_2_- HMSN and stirred to obtain the NH_2_-HMSN@CA composites.

### 2.3. Preparation of Nanocellulose-Based Films

Firstly, the 50 mL nanocellulose solution with a solid content of 0.5% was prepared, referred to as CNF. Next, NH_2_-HMSN@CA, corresponding to 10% of the nanocellulose solid content, was added and the mixture was stirred for 20 min to form the CAF solution. Finally, anthocyanin, corresponding to 2.5%, 5%, and 10% of the nanocellulose solid content, was added and the mixture was stirred for an additional 20 min to obtain the CBF solution. Each of these solutions was then poured into polytetrafluoroethylene (PTFE) plates and dried at 35 °C for 24 h to form the CNF, CAF, and CBF films, respectively. After drying of the CBF films, the ratio of nanocellulose, NH_2_-HMSN@CA, and anthocyanin was 20:2:1.

### 2.4. Characterization of Nanoparticles and Films

The microstructure of NH_2_-HMSN@CA and the films was analyzed by scanning electron microscopy (SEM, Zeiss Sigma 360, Oberkochen, Germany). Fourier transform infrared spectrometer (FT-IR,) with a scanning range of 4000–400 cm^−1^, was used to analyze the chemical bonding of NH_2_-HMSN@CA and the films. The zeta potential of the samples was determined using a Malvern Zetasizer Nano ZS90 (Malver Panalitycal, Worcetershire, UK). X-ray diffraction spectroscopy (XRD, Bruker D2 Phaser, Germany) with a 2θ scanning range of 5°–90° for CuKα radiation was used to analyze the crystal structure of the samples. An X-ray photoelectron spectrometer (XPS, Thermo Scientific K-Alpha, Waltham, MA, USA) was used to analyze the elements of the composite films. Tensile strength and elongation at break were measured using an INSTRON 5565 (Norwood, MA, USA) tensile/compression testing machine. The testing length was 25 mm, and the tensile speed was set to 10 mm/min. For each sample film, three or more parallel specimens were tested, and the results were averaged.

### 2.5. Dissolution Rate and Swelling Rate

The swelling and dissolution rates were determined following the method described by Wu et al. [34]. First, the dried film sample was weighed (M0). Then, the sample was completely immersed in a centrifuge tube containing 25 mL of distilled water and maintained in a constant temperature and humidity chamber for 24 h. Afterward, the sample was removed with surface water absorbed using filter paper, then weighed (M1). Finally, the sample was dried in an oven at 40 °C until a constant weight was achieved and weighed again (M2). The dissolution rate (DR) and swelling rate (SR) were calculated as follows:

(1) $$DR=\frac{M0-M2}{M0}\times 100$$

(2) $$SR=\frac{M1-M0}{M0}\times 100$$

### 2.6. In Vitro Release Test

The cumulative release rate of cinnamaldehyde was measured by the dialysis membrane method [35]. The prepared NH_2_-HMSN@CA (10 mL) was transferred into a dialysis bag (molecular weight cut off 3500D). The bag was suspended in phosphate buffered saline (PBS, 0.01 mol/L, pH 7.2) at room temperature and stirred at 200 r/min. At each of the predetermined time points, the test samples (2 mL) were taken to detect the absorbance of cinnamaldehyde (Ai) using UV-Vis spectrophotometry (UV-1600 type, Shanghai, China). Then, the cinnamaldehyde content was calculated by using the standard curve equation of cinnamaldehyde (y = 0.1979x − 0.0152, r² = 0.9992).

### 2.7. Antibacterial Experiments

The suspensions of *E. coli* and *S. aureus* were diluted and inoculated onto Luria–Bertani (LB) agar plates. The suspensions of *E. coli* and *S. aureus* were mixed with sterilized films and LB liquid medium, followed by incubation at 37 °C for 10 h. The mixture was then serially diluted by 10^5^ times and inoculated onto LB agar plates to observe the colony growth and the colonies were counted to calculate the antibacterial rate. The inoculum size of *E. coli* and *S. aureus* for the inhibition circle and co-culture experiments was 10^5^ CFU/mL.

(3) $$Antibacterial\;rate\;\left(\%\right)=\frac{A-B}{A}\times 100$$

In the equation above, A represents the average number of colonies (CFU) in the control group, and B represents the average number of colonies (CFU) in the treatment group.

### 2.8. Colorimetric Experiment

The chromaticity values of the films were tested using a colorimeter. The overall color difference was represented by ΔE, and its calculation is shown in Equation (4). Each sample was tested at no less than 3 points, and the result was taken as the average value.

In the formula, L_0_, a_0_, and b_0_ are the chromaticity values of the standard white (L_0_ = 97.13, a_0_ = −0.04, b_0_ = −0.06).

The prepared CBF film was cut into 2 × 2 cm squares, which were immersed in phosphate buffered saline (PBS, 0.01 mol/L, pH 7.2) with pH values ranging from 2 to 12. Photographs were taken every 2 min using a camera to record and compare the color changes until the color of the indicator film stabilized. A colorimeter was used to measure the chromaticity values of the film, and the color difference of the stabilized indicator film was calculated using Equation (4).

(4) $$∆E=\sqrt{{\left(L-L_{0}\right)}^{2}+{\left(a-a_{0}\right)}^{2}+{\left(b-b_{0}\right)}^{2}}$$

In the formula, L_0_, a_0_, and b_0_ represent the initial chromaticity values of the film, while L, a, and b represent the chromaticity values of the film after color change.

### 2.9. Statistical Analysis of Data

All experiments were performed in three biological replicates, and all data were presented as mean ± standard error (SE). IBM SPSS Statistics 27 and Origin 2024 were applied to analyze the data. The value of *p* < 0.05 was considered statistically significant.

## 3. Results and Discussion

### 3.1. Characterization of Nanoparticles

**Figure 1:**
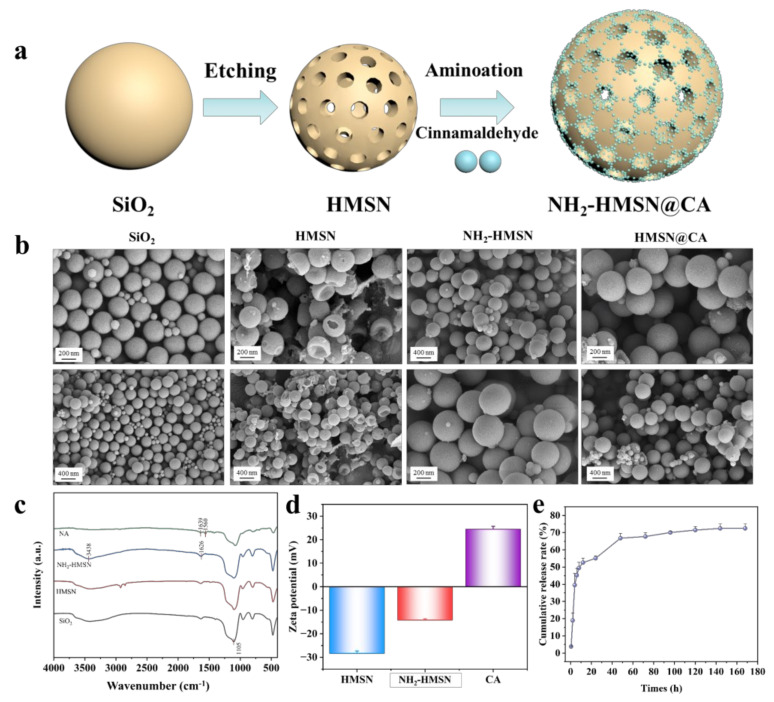
Schematic illustration of the preparation process of NH_2−_HMSN@CA (**a**), SEM (**b**), FTIR spectra (**c**), zeta potential (**d**), and cumulative release rate (**e**) of NH_2−_HMSN@CA. NA: NH_2−_HMSN@CA (**c**).

Firstly, aminosylated silica-supported cinnamaldehyde preservative (NH_2_-HMSN@CA) was synthesized. In this method, hollow mesoporous silica nanoparticles were etched from the prepared silicon spheres and then modified with the amino group. The purpose of this process was to increase the loading rate of cinnamaldehyde by connecting cinnamaldehyde with the amide bond and further loading cinnamaldehyde in the cavity formed by the hollow mesoporous structure. (Figure 1a). As shown in Figure 1b, the SEM images clearly show the morphological structures of both pristine and modified silica. The initially synthesized silica spheres exhibited smooth surfaces, whereas the MSNs formed by surface etching displayed a roughened texture. The HMSNs had a distinct hollow structure with uniformly spherical particles and no aggregation, and after amination and loading with cinnamaldehyde, the surface of the spheres became rough, suggesting that cinnamaldehyde was successfully loaded. Subsequently, the FT-IR analysis further confirmed the successful incorporation of both the amino groups and cinnamaldehyde (Figure 1c). As can be seen from the figure, the Si-O characteristic peak of silica appeared at 1105 cm^−1^. After amino modification, a new N-H peak appeared near 1626 ^−1^, along with an enhanced peak at 3438 ^−1^ When cinnamaldehyde was introduced to the modified hollow mesoporous silica, a C=N peak emerged at 1639 ^−1^, and the peak around 3438 ^−1^ disappeared due to the formation of a Schiff base [36,37]. In addition, zeta potential analysis provided further evidence of the successful synthesis of the NH_2_-HMSN@CA preservative (Figure 1d). The introduction of amino groups increased the surface potential due to the added positive charges. After loading with cinnamaldehyde, the potential increased further. This was likely due to changes in surface charge distribution when cinnamaldehyde bound to the amino-modified mesoporous silica, possibly causing additional interactions with ions or water in the surrounding environment [38].

Furthermore, to evaluate the release behavior of the antibacterial agent, a release experiment was conducted (Figure 1e). As can be seen in the figure, the release process occurred in two distinct phases. First, the initial burst release phase, where approximately 54% of the cinnamaldehyde was rapidly released within 24 h, likely driven by the concentration gradient between the internal and external solutions, facilitating fast diffusion. Second, the subsequent sustained release phase, where the cumulative release increased gradually from 54% to 72% between days 2 and 7. The sustained release is attributed to two main mechanisms: First, cinnamaldehyde reacts with the amino groups on the HMSN surface to form relatively stable C=N bonds, which gradually hydrolyze under acidic conditions or in the presence of moisture, leading to a slow release of cinnamaldehyde [39]. Second, the regular pore structure and high specific surface area of the hollow mesoporous silica provide a physical barrier that slows the diffusion of cinnamaldehyde molecules, thereby limiting the release rate. These mechanisms address the challenges associated with cinnamaldehyde’s volatility, instability, and strong odor, thereby enhancing its potential application in preservation materials.

### 3.2. Physicochemical Properties of the Films

**Figure 2:**
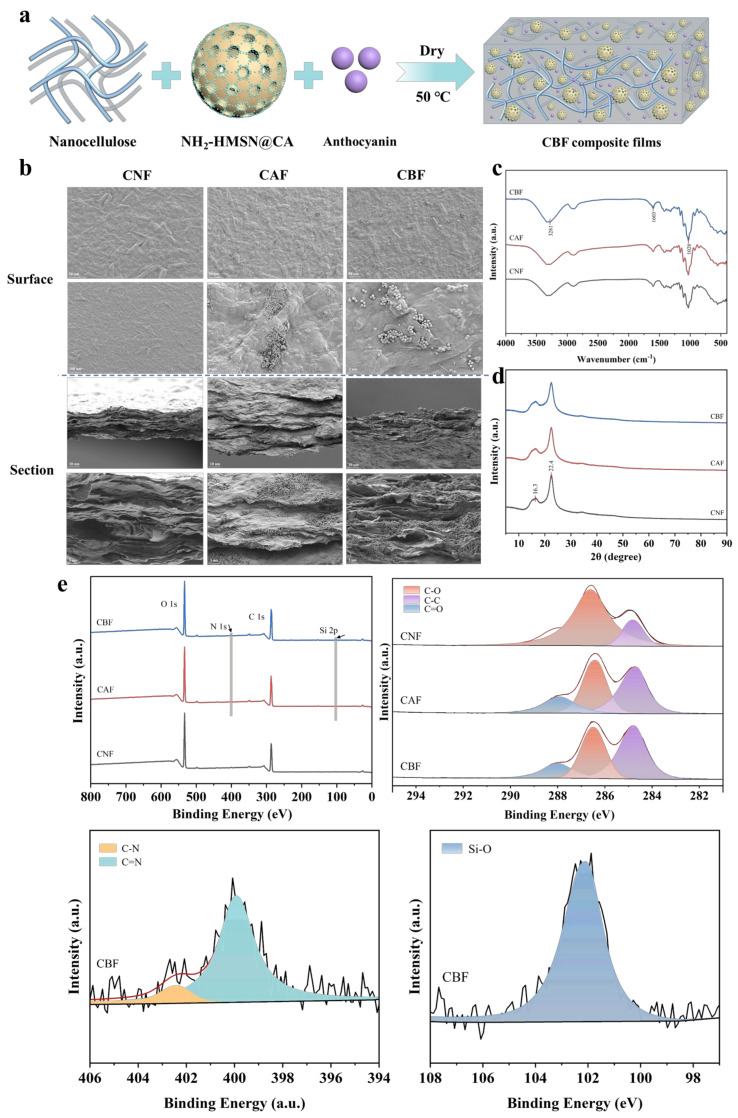
Schematic illustration of the preparation process of films (**a**), SEM images (**b**), FTIR spectra (**c**), XRD spectra (**d**), and XPS spectra (**e**) of composite films.

Subsequently, using the synthesized NH_2_-HMSN@CA as the antibacterial agent and blueberry anthocyanin as the chromogenic agent, the composite films were prepared by combining with nanocellulose, which could not only be preserved but also monitored (Figure 2a). The surface and cross-section of the films were analyzed by SEM to observe their internal morphologies and structures (Figure 2b). As shown in the figure, the CNF film showed a distinct fibrous network on the surface with interwoven fibers, and its cross-section exhibited a layered structure with porosity. Upon the addition of NH_2_-HMSN@CA, the spherical HMSN particles filled the pores, resulting in a more uniform surface and a denser layered structure. This densification was likely due to the multi-dimensional interwoven interaction and electrostatic adsorption between the nanocellulose and HMSN. However, the film surface became rougher with the further addition of blueberry anthocyanins, displaying noticeable aggregated particles. The reason for this may be that the addition of blueberry anthocyanins disrupted the interaction forces between nanocellulose and modified silica, but the film might also have benefited from the enrichment of spoilage substances, which is conducive to the colorimetric response. Afterwards, the chemical structures of the films were analyzed by FT-IR (Figure 2c). The characteristic peaks of nanocellulose at 3281 ^−1^ and 1603 ^−1^ corresponded to the -OH and C=O stretching vibrations, respectively. Additionally, upon the introduction of NH_2_-HMSN@CA, Si-O bonds were observed, contributing to the strengthening of the peak at 1028 ^−1^ After NH_2_-HMSN reacted with cinnamaldehyde to form imine bonds (-CH=N-) via a Schiff base reaction, the absorption peak at 1603 ^−1^ was enhanced. In the CBF film, the introduced blueberry anthocyanins, which contained -OH groups, resulted in a narrowing and intensification of the -OH absorption peak, while the aromatic rings in the anthocyanins further enhanced the peak at 1603 ^−1^ These analyses confirm the successful incorporation of both the antibacterial agent and the colorimetric agent into the films. Furthermore, as shown in the XRD patterns of the CNF film (Figure 2d), the nanocellulose exhibited characteristic peaks at 2θ = 22.4° and 2θ = 16.3°. In the CAF samples, the characteristic peak at 2θ = 22.4° showed noticeable broadening, which could be attributed to the formation of a certain encapsulation structure between NH_2_-HMSN@CA and nanocellulose on the surface. In the CBF films, no new diffraction peaks emerged, indicating that both NH_2_-HMSN@CA and blueberry anthocyanins were uniformly dispersed within the nanocellulose matrix without large-scale aggregation. XPS spectra were used to analyze the chemical bonds in the films (Figure 2e). In the overall XPS spectrum, CNF mainly contained C and O elements. With the introduction of NH_2_-HMSN@CA in CAF, the elements N and Si appeared. After the addition of anthocyanin (CBF), the elements remained unchanged. Next, we analyzed the C, O, and N spectra. In the C spectrum, the CNF contained three types of chemical bonds—C-C, C-O, and C=O—with binding energies located at 284 eV, 285 eV, and 287 eV, respectively. We used oxidized CNF containing C=O, although in lower amounts. In CAF, the proportion of C=O increased significantly due to the incorporation of NH_2_-HMSN@CA, as cinnamaldehyde contained -CHO groups. The binding energies of C-O and C=O increased, and the peaks shifted to higher binding energies, at 286 eV and 288 eV, respectively. This could be due to the formation of hydrogen bonds or electron transfer between NH_2_-HMSN@CA and CNF, which led to a decrease in electron density around C-O and C=O. In CBF, the peaks for C-O and C=O shifted further to the left, and the proportion of C=O increased. This could be due to the addition of blueberry anthocyanin, which was rich in phenolic hydroxyl groups and could form π-π stacking or hydrogen bonds with CNF. In the O spectrum, C-O (531 eV), C=O (533 eV), and C-OH (532 eV) are the three typical chemical bonds of oxidized CNF. In CAF, the proportion of C=O increased, which was attributed to the rich aldehyde groups in cinnamaldehyde encapsulated by HMSN. In CBF, the proportions of both C=O and C–OH increased, as blueberry anthocyanin contained phenolic hydroxyl groups. The results of the O spectrum were consistent with those of the C spectrum. In the N spectrum, in CBF, the proportion of C=N at 399 eV was significant, indicating that NH_2_-HMSN@CA was successfully incorporated into the CNF film and that cinnamaldehyde was successfully introduced through imine bonds. The chemical bonds identified by XPS were consistent with those observed in the FTIR analysis, jointly confirming the successful synthesis of the composite film. This comprehensive characterization demonstrates that the integrated antibacterial and colorimetric film has been successfully prepared, with uniform dispersion and the intended chemical interactions among its components [40].

**Figure 3:**
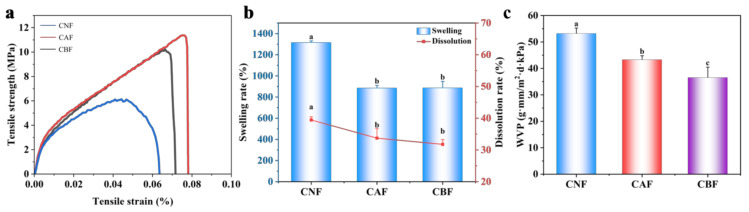
Mechanical properties (**a**), swelling and dissolution rate (**b**), and water vapor permeability (**c**) of composite films. The different letters indicate statistically significant differences at the level of *p* < 0.05.

Moreover, we evaluated the physical properties of the films to demonstrate their potential for food packaging applications. The mechanical performance of the films was analyzed using stress–strain curves. As illustrated in Figure 3a, the pure nanocellulose film exhibited the poorest overall performance. However, upon incorporating NH_2−_HMSN@CA, both the tensile strength and elongation at break increased, indicating improved toughness and the ability to withstand larger deformations without fracturing. This enhancement could be attributed to the interactions between HMSN and the nanocellulose, along with the filling of pores, which resulted in a denser structure. Although the mechanical properties slightly decreased after the incorporation of blueberry anthocyanins, they still exhibited better performance compared with pure nanocellulose. These observations were consistent with the more compact morphology observed in the SEM images. Additionally, as can be seen from Figure 3b, CNF films had a high solubility and swelling rate in water, leading to instability under humid conditions. In contrast, the multifunctional composite films containing the antibacterial agent and anthocyanin exhibited a lower solubility and swelling rate, reflecting an enhanced environmental stability of the composite film. This improved water resistance was likely due to the denser structure of the composite film, which imparted its better barrier properties. Because maintaining food freshness and extending shelf life greatly depend on the barrier properties of the packaging [41,42], the water vapor and oxygen barrier properties of the films were further discussed. As shown in Figure 3c, the WVP of CNF was 53 g·mm/m^2^·d·kPa, while those of CAF and CBF were only 43 and 36 g·mm/m^2^·d·kPa, respectively. The incorporation of an appropriate amount of nanomaterials reduced the water vapor permeability of the composite film. The composite film significantly reduced the ingress of external moisture into the packaging, primarily because the electrostatic interactions between HMSN and nanocellulose resulted in a more compact and dense structure. This confirms that integrating HMSN and anthocyanins into the nanocellulose matrix can effectively lower the water vapor transmission rate [43]. Overall, the nanocellulose-based composite films we developed exhibited superior mechanical properties and barrier performance compared with pure nanocellulose films. These improvements suggest that the composite film has significant potential for use in food packaging applications [44].

### 3.3. Antibactirial Properties

**Figure 4:**
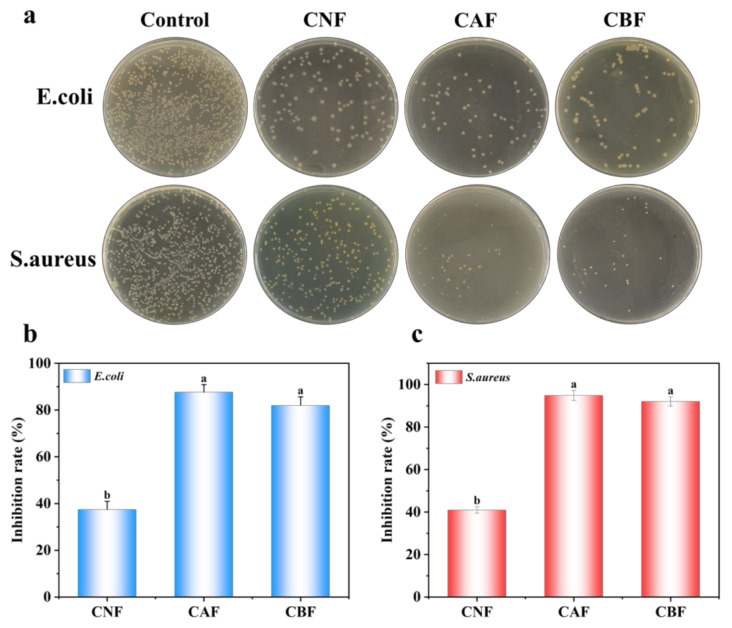
Antibacterial properties of composite films. Co-culture assay (**a**); inhibition rate of *E. coli* (**b**); and inhibition rate of *S. aureus* (**c**). The different letters indicate statistically significant differences at the level of *p* < 0.05.

To evaluate the antibacterial performance of the composite films, tests were conducted against *E. coli* and *S. aureus* (Figure 4a,b). As shown in Figure 4a, for both bacterial strains, CNF only slightly inhibited colony growth. However, after the addition of NH_2−_HMSN@CA, a significant reduction in bacterial growth was observed. The subsequent incorporation of anthocyanins did not compromise the film’s antibacterial properties. Both the CAF and CBF films exhibited excellent antibacterial activity, with growth inhibition rates exceeding 80% for *E. coli* and over 90% for *S. aureus*. The primary antibacterial active factor originates from the loaded cinnamaldehyde [45]. This is because, when exposed to an aqueous environment, the imine bonds formed between cinnamaldehyde and -NH_2_ groups undergo slow hydrolysis, gradually releasing cinnamaldehyde. In addition, the cinnamaldehyde adsorbed within the hollow structure of HMSN is released in a controlled manner, as demonstrated by the cumulative release experiments (Figure 1e). The released cinnamaldehyde then interacts with the bacteria, effectively inhibiting their growth and reproduction. Notably, cinnamaldehyde exhibits a higher antibacterial efficacy against Gram-positive bacteria. This difference is likely due to the cell wall composition: Gram-positive bacteria have cell walls mainly composed of peptidoglycan, which can be more readily disrupted by essential oils, whereas the outer membrane of Gram-negative bacteria, rich in lipopolysaccharides, impedes the entry of hydrophobic substances like essential oils [46,47]. Overall, the results confirm that the CBF film has excellent bactericidal properties, making it a promising material for antibacterial applications in food packaging [48].

### 3.4. pH Sensitivity Test

**Figure 5:**
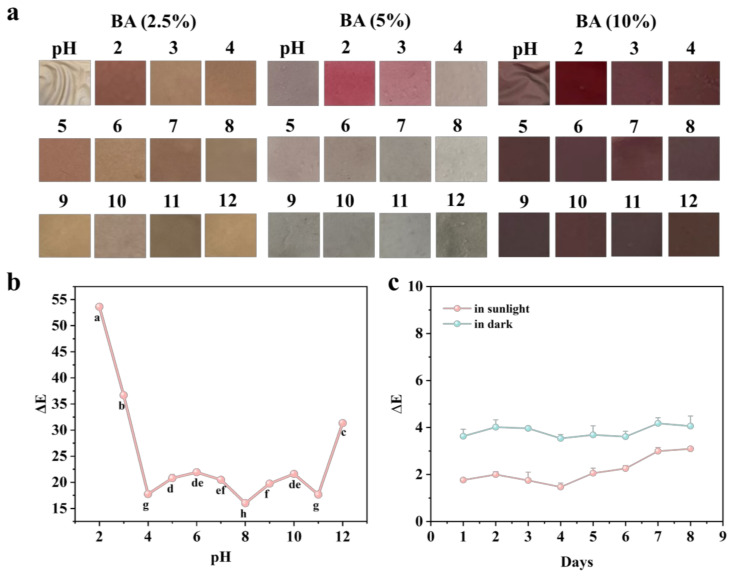
Color change of the indicator films at different concentrations at different pH (**a**); change in color difference value of the indicator films at different pH (**b**); stability of the indicator films (**c**). The different letters indicate statistically significant differences at the level of *p* < 0.05.

Blueberry anthocyanins exhibit different colors under varying pH conditions due to the presence of distinct ionic species, which meets the requirements for intelligent packaging to visually monitor changes in food quality [31]. In this study, blueberry anthocyanins were incorporated into a nanocellulose film, and the film’s color changes induced by pH variations during food storage were employed to assess food quality changes [32]. As depicted in Table 1, the prepared CBF film initially displayed a distinct purple color, the characteristic color of blueberry anthocyanins, while the CAF film showed a white color and the CAF film showed a yellow color. Subsequently, the films with different anthocyanin concentrations were immersed in solutions with varying pH values, and their color changes were observed, as shown in Figure 5a. We found that the film containing 2.5% anthocyanin exhibited minimal color change, appearing slightly yellow. This effect may be attributed to the low amount of anthocyanin undergoing oxidation and structural alteration during heating, resulting in poor color development. In contrast, the film with 5% anthocyanin demonstrated significant color changes within 5 min; it turned pink under acidic conditions, became lighter under neutral conditions, and shifted to gray or blue under alkaline conditions. The color difference was pronounced and observable by the naked eye, which can be ascribed to the uniform and dense structure of the film. When the anthocyanin concentration was increased to 10%, the color change was slower. Moreover, the film remained purple for 20 min and even showed signs of decolorization. This phenomenon might result from excessive anthocyanin leading to aggregation and interactions with nanocellulose, thereby slowing down the color change. Therefore, in our experiments, a 5% anthocyanin concentration yielded the most significant and rapid color development (Figure 5b).

Subsequently, color stability tests were conducted. As shown in Figure 5c, the film maintained a stable color for 8 days, without significant color differences at room temperature. However, under light exposure, the color difference increased. This may be due to the sensitivity of anthocyanins to light, where prolonged exposure triggers oxidation and decomposition reactions. These reactions can disrupt the conjugated system in anthocyanin molecules, altering the absorption spectrum and consequently the color. In summary, the composite film developed in this study not only exhibits excellent mechanical, barrier, and antibacterial properties but also shows distinct color variations, offering dual functionality for preservation and monitoring and holding promising potential for future applications.

## 4. Conclusions

In this study, hollow mesoporous silica loaded with cinnamaldehyde was successfully synthesized, and then mixed with blueberry anthocyanins and nanocellulose to prepare the composite film with antibacterial and indicator functions (CBFs). During this process, the prepared amino silica had an excellent regulatory effect on the release of cinnamaldehyde through the dynamic Schiff base reaction. Furthermore, the film exhibited excellent antibacterial performance against *E. coli* and *S. aureus*, and also possessed good mechanical properties and water vapor permeability. With the addition of 5% blueberry anthocyanins, the film showed an excellent colorimetric response to pH changes. Therefore, the CBF film can be applied to fruits, vegetables, seafood, and meat products, offering both antibacterial effects and real-time monitoring of food quality changes, showing great potential in intelligent food packaging.

## Figures and Tables

**Table 1:** Chromaticity values of CNF, CAF, and CBF films.

	L	a	b	∆E	Film Color
CNF	82.87	−0.22	−0.28	14.26	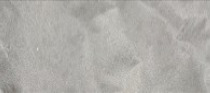
CAF	80.42	−2.10	7.74	18.56	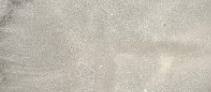
CBF	33.01	3.01	−4.31	64.33	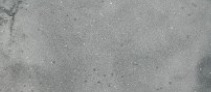

## Data Availability

The original contributions presented in this study are included in this article, further inquiries can be directed to the corresponding author.
